# Laser Fabrication and Performance of Flexible Pressure Sensors with Ridge-Mimicking Spatially Ordered Microstructures

**DOI:** 10.3390/mi15121441

**Published:** 2024-11-28

**Authors:** Linjing Wu, Chao Gao, Jincheng Wang, Chen Zhang, Yuzhi Ke

**Affiliations:** 1 Pen-Tung Sah Institute of Micro-Nano Science and Technology, Xiamen University, Xiamen 361102, China; wulinjing@xmu.edu.cn (L.W.);; 2 Shenzhen Research Institute of Xiamen University, Xiamen University, Shenzhen 518000, China

**Keywords:** flexible pressure sensor, microstructures, laser fabrication, piezoresistivity

## Abstract

The proliferation of flexible pressure sensors has generated new demands for high-sensitivity and low-cost sensors. Here, we propose an elegant strategy to address this challenge by taking a ridge-mimicking, gradient-varying, spatially ordered microstructure as the sensing layer, with laser processing and interdigitated electrodes as the upper and lower electrode layers. Simultaneously, the entire structure is encapsulated with polyimide (PI) tape for protection, and the fabrication process is relatively feasible, facilitating easy scaling. The presented results show that the flexible pressure sensor exhibits a sensitivity of 1.65 kPa^−1^ across a pressure range of 0 to 1100 kPa. Furthermore, the sensor displays low hysteresis, as well as rapid response and recovery times of 62 ms and 83 ms, respectively. Finally, we demonstrate the application potential of the sensor for monitoring joint movements, especially for detecting pressure and direction in finger joints. This technology shows great potential for applications in smart robotics, wearable devices, health monitoring, and other emerging technologies.

## 1. Introduction

Flexible pressure sensors, as the indispensable components in wearable electronic devices, are becoming increasingly prominent in the fields of smart robotics, vital signs monitoring, wearable electronic devices, and human–machine interactions due to their compact size, light weight, excellent flexibility, and strong adhesive properties, allowing them to maintain intimate conformal contact with human skin. Therefore, they comprise the current research frontier and are hotspots for use as intelligent sensors [1]. Flexible pressure sensors based on capacitive [2], piezoresistive [3], piezoelectric [4], and triboelectric [5] properties are commonly used to convert pressure information into electrical signals. The underlying mechanism of these sensors is to convert mechanical deformation caused by pressure into electrical signals, such as changes in capacitance, resistance, or current. Among them, piezoresistive sensors have the advantage of a simple structure, low fabrication costs, and ease of signal acquisition; thus, they have been widely used in pressure detection applications.

Graphene is frequently used as a conductive material in the dielectric layer of sensors because of its excellent optical, electrical, and mechanical properties. However, its performance in practical applications is often affected by temperature fluctuations. To counteract this issue, P-type doping is used to develop near-zero resistance graphene films, effectively reducing temperature interference [6]. Nevertheless, graphene-based planar structures tend to exhibit insufficient deformation under very low pressures, resulting in limited detectable changes in resistance, which hinders the sensitivity of the sensors. Introducing microstructures into the dielectric layer has proven to be an effective approach to enhance the sensitivity of flexible sensors [7].

Various methods have been explored to create microstructures with different shapes, such as microspheres, micropillars, microcones, and microgrooves [8]. These microstructures create air gaps within the dielectric layer, which can significantly reduce elastic resistance and increase deformation under applied stress, thereby inducing a stronger electrical signal response. This approach represents a promising avenue for advancing the development of flexible pressure sensors.

Microstructure fabrication methods typically include templating [2], self-assembly [9], nanoimprinting [10], ion etching [11], and photolithography [12]. Among these, templating is the most widely used method for transferring microstructures onto polydimethylsiloxane (PDMS) films. However, it faces challenges such as low fabrication precision and limited mold reusability. While self-assembly and nanoimprinting can produce intricate microstructures, these methods require high technical expertise and involve complex processes, increasing fabrication costs. Similarly, ion etching requires specialized equipment, making it less suitable for small-scale production. Photolithography, which is ideal for microstructures on silicon-based materials, often involves complex steps like mask alignment and exposure, adding to the overall complexity. Up until now, developing highly sensitive and low-cost flexible pressure sensors has remained a significant challenge [13,14,15].

Herein, to overcome the above limitations, we developed a near-zero resistance graphite power, carbon nanotube, polydimethylsiloxane (GP/CNT/PDMS) conductive composite to serve as the dielectric layer. Through direct laser writing, we fabricated a pressure-sensitive layer with gradient-varying microstructures using a layer-by-layer scanning approach. The sensor was then encapsulated with interdigitated electrodes and PI film. Finally, we conducted a comprehensive investigation into its working principles, performance, and potential applications.

## 2. Materials and Fabrication Method

### 2.1. Experimental Materials and Instruments

The relevant raw materials used in this work to fabricate the flexible pressure sensor are shown in Table 1. The commercial instruments for preparing the sensing layer and electrode are listed in Table 2.

### 2.2. Fabrication Process of Flexible Pressure Sensor

#### 2.2.1. Ridge-Mimicking Graphene Dielectric Layer

(1) Preparation of GP/CNT/PDMS conductive composite

**Figure 1:**
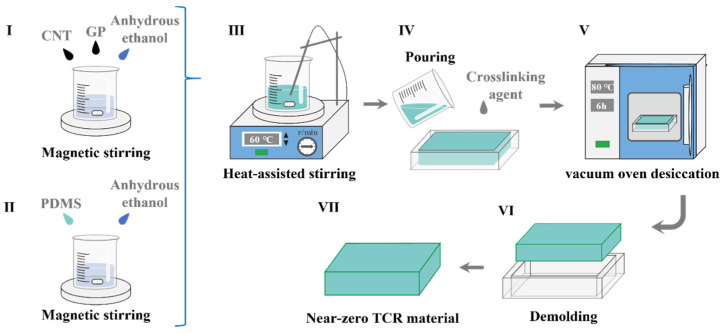
Preparation process of GP/CNT/PDMS conductive composite (I Preparation of the electronic solution, II Preparation of the PDMS, III Mixture of the PDMS and electronic solution, IV GP/CNT/PDMS molding, V Heating for curing, VI Demolding of the GP/CNT/PDMS, VII As-prepared temperature coefficient of resistance (TCR) material).

The GP/CNT conductive mixture was prepared by ultrasonically mixing graphite powder (GP, 0.57 g), which exhibits excellent conductivity and a large aspect ratio, with multi-walled carbon nanotubes (CNT, 0.3 g) at a ratio of 0.95:1. Anhydrous ethanol (120 mL) was used as the solvent. The mixture was then combined with 14 g of PDMS precursor solution, which is chemically stable, highly flexible, and easy to process, dissolved in 20 mL of anhydrous ethanol. This mixed solution was heated and stirred at 333.15 K with a stirring speed of 500 r/min until homogeneous and then dried. After drying, a curing agent was added to the material, and the mixture was stirred before being pressed into a mold. The composite was cured by vacuum drying at 353.15 K for 10 h. Finally, the cured GP/CNT/PDMS conductive composite was demolded. The detailed preparation process is shown in Figure 1.

(2) Laser processing of GP/CNT/PDMS conductive composite

**Figure 2:**
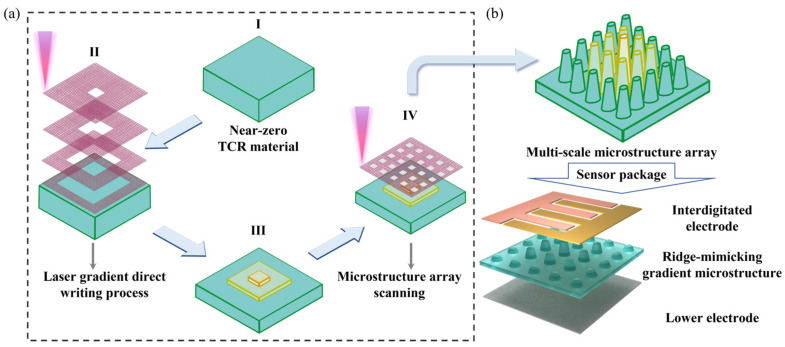
(**a**) Fabrication process of the ridge-mimicking spatially ordered microstructure. (I As-prepared TCR material, II Fabrication process of the ridge-mimicking microstructure, III As-prepared ridge-mimicking microstructure, IV Fabrication process of the spatially ordered microstructure) (**b**) Assembly process of the flexible pressure sensor.

Under ultraviolet laser pulses, the surface of the GP/CNT/PDMS conductive composite absorbs the laser energy, causing the temperature to rise, resulting in material melting and vaporization. As the laser beam moves across the surface, the heated areas cool and solidify, forming micropillar structures. With an increasing number of laser scans, these micropillars gradually transition into ridge-mimicking structures. By controlling the scanning speed, conductive microcones of varying heights can be obtained. The detailed fabrication process of the ridge-mimicking gradient structures is displayed in Figure 2a. Meanwhile, the different microstructure optimizations under different microstructure heights and arrangements are discussed in Figures S1–S3, and the optimized microstructure array (3 × 3 array) under 1000 mm/s is obtained. Specifically, under the processing parameters of a 20 kHz laser frequency, five scans, and a scanning speed of 1000 mm/s, a gradient microstructure is formed through laser machining along different grid patterns. Subsequently, an additional 10 scans were performed using a cone-patterned grid, eventually resulting in a gradient array of conductive microcones. Finally, the assembly process of the flexible pressure sensor based on the ridge-mimicking gradient structures is shown in Figure 2b.

#### 2.2.2. Sensor Package

**Figure 3:**
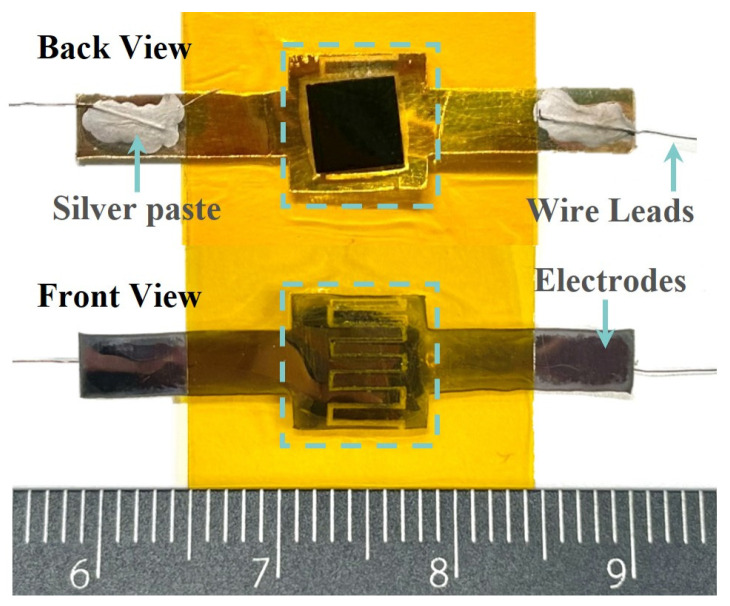
Optical image of the ridge-mimicking spatially ordered microstructure sensor.

The sensor was encapsulated by first sputtering 20 nm of titanium and 40 nm of gold onto a cleaned and dried polyethylene terephthalate (PET) film using a magnetron sputtering machine (DONGAN, EXPLORER-14, Detroit, USA). Laser patterning was then applied to form interdigitated electrodes. To protect the device from external environmental factors such as humidity and temperature and to enhance its stability, the sensor was sealed using PI tape [16,17]. This process resulted in the formation of an ordered multiscale piezoresistive sensor, as shown in Figure 3.

### 2.3. Performance Testing of Flexible Pressure Sensor

The current version of the sensor was measured using a digital multimeter (Keithley2400, Tektronix, Beaverton, OR, USA), allowing us to evaluate its sensitivity, hysteresis, repeatability, stability, response time, and pressure detection limits. During testing, a high-precision stepper motor (FuYu FSK40, Chengdu, China) was used to apply pressure to the ridge-mimicking ordered conductive microcone array sensor. A commercial pressure gauge (Hengyuan HYBH-101, Yangzhou, China) was used to record the applied pressure. The sensor was connected to the digital multimeter using conductive tape, with a constant voltage applied. The relationship between the applied pressure and the relative change in current was recorded. Additional tests were conducted to measure hysteresis, fast response time, and recovery time.

## 3. Results and Discussion

### 3.1. Design Strategy of the Flexible Pressure Sensor

**Figure 4:**
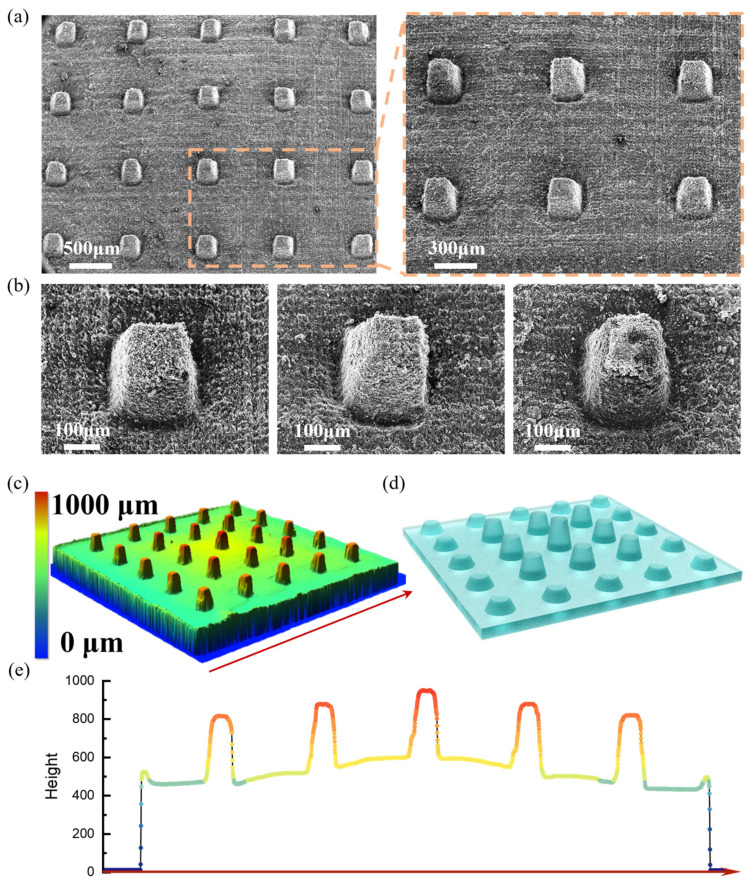
(**a**) SEM image of the conductive microstructure array (3 × 3 array). (**b**) SEM image of the tertiary gradient microcone structure. (**c**) Microstructural ultra-deep field image of the sensing layer after fabrication. (**d**) Schematic of the microstructure design of the sensing layer. (**e**) Height variation in the middle section of the microstructure array ultra-deep field image scanning data.

**Figure 5:**
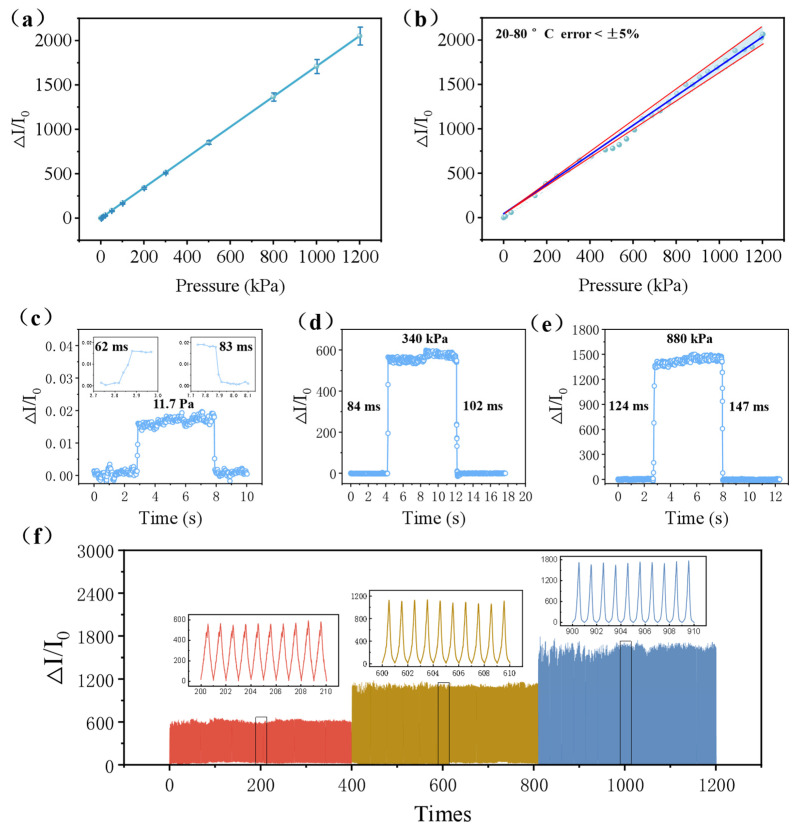
(**a**) Sensitivity testing curve of the ridge-mimicking spatially ordered flexible pressure sensor at different pressures. (**b**) Temperature testing on sensor sensitivity. Response and recovery time of the sensor at (**c**) 11.7 Pa, (**d**) 340 kPa, and (**e**) 880 kPa. (**f**) Stability testing of the sensor at different times.

For applications in human–machine interactions and wearable electronics, it is essential to achieve rapid responses to small forces, necessitating the development of highly sensitive flexible sensors. To develop a high-sensitivity flexible pressure sensor with rapid response, a ridge-mimicking structure with gradient-varying microstructures is developed in this work, as shown in Figure 4a. This design features conductive microcones with a narrow top and wide base, arranged in a ridge-like pattern, with a gradient that decreases from the center of the stress concentration outward. Figure 4b–e shows that the microcone structures with a tertiary gradient are efficiently constructed after laser processing, and the rich surface microstructures help to improve the sensitivity of the structural deformation. Since air offers negligible resistance to deformation, this structural design significantly enhances the compressibility of the device, enabling a relatively large change in the contact area under small pressure. Additionally, the conductive microcone structure creates both surface and bulk conduction pathways between the pressure-sensitive layer and the electrodes, significantly amplifying the electrical signal. Furthermore, the ridge-mimicking microcone structure allows the device to maintain a relatively uniform change in contact area under stress concentration, ensuring high sensitivity, as shown in Figure 5.

### 3.2. Performance Investigation of the Ridge-Mimicking Spatially Ordered Flexible Pressure Sensor

Figure 5a illustrates the sensitivity of the flexible pressure sensor with ridge-mimicking microstructures, indicating that the high sensitivity of the sensor at different pressures can be achieved. Additionally, the mathematical equations for sensitivity can be obtained by linear fitting. The specific equation is expressed as follows:

y=1.65x−0.0053

From this equation, the pressure sensitivity of 1.65 kPa^−1^ and the R^2^ value of 0.996 were achieved over a pressure range of 1 to 1100 kPa.

On the other hand, the sensitivity error of the as-prepared sensor was less than 5% at temperatures ranging from 20 °C to 80 °C, indicating that the sensors exhibit excellent thermal stability, as shown in Figure 5b. This also helps to extend the use of the sensors under different temperature conditions. To evaluate the response and recovery time of the sensors, different objects were placed directly on top of the sensor and then rapidly removed after the signal stabilized. The test results are presented in Figure 5c–e, indicating that the response time is 62 ms, and the recovery time is 83 ms under low pressure (11.7 Pa). This is because upon applying pressure to the dielectric layer, the central and tallest microcone of the ridge-mimicking spatially ordered structure deforms first, transferring the force to the upper electrode and subsequently distributing it across the gradient-varying microcones. More importantly, this allows the deformed microcones to return to their original shape more quickly, significantly improving the sensor’s response and recovery times. Additionally, as the pressure increases, the response time and recovery time of the sensors also increase (Figure 5d,e). To evaluate the sensor’s stability, pressures of 300 kPa, 600 kPa, and 900 kPa were applied for 400 consecutive cycles, as illustrated in Figure 5f. The results show that the sensor consistently performed at different pressure levels and during 400 consecutive cycles, demonstrating excellent stability.

**Figure 6:**
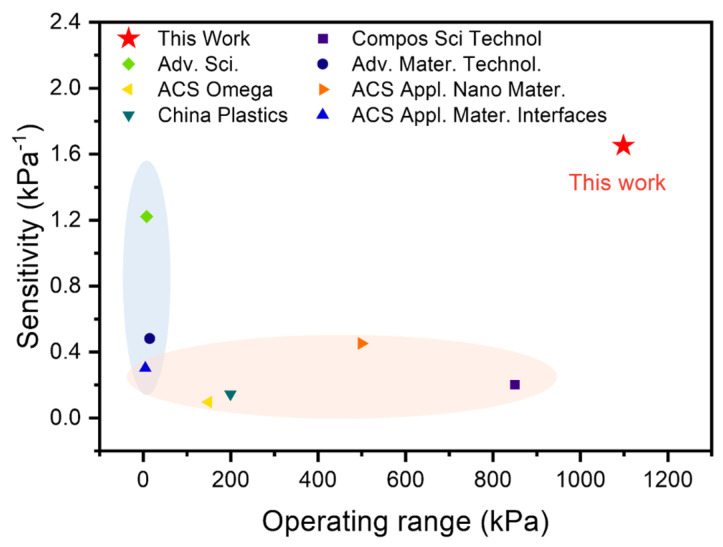
Performance comparison between the work in this paper and that of related works [12,18,19,20,21,22,23].

Furthermore, we compared the performance of the piezoresistive sensor by using different manufacture methods, as shown in Figure 6. Compared to the other sensors, the ridge-mimicking spatially ordered flexible pressure sensor exhibits high sensitivity and fast response times. This fabrication method is simple and offers stable, controllable fabrication, making the sensor highly promising for applications in wearable electronics and health monitoring. More detailed results are summarized in Table 3.

### 3.3. Applications of the Sensors

**Figure 7:**
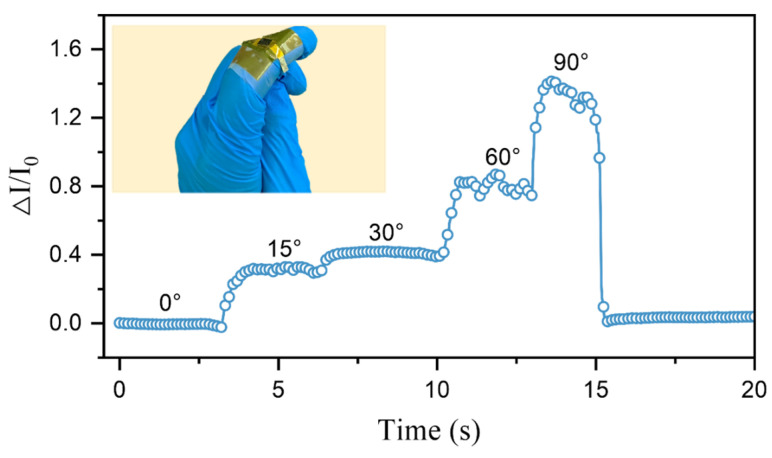
Schematic of flexible pressure sensor in finger bending test.

**Figure 8:**
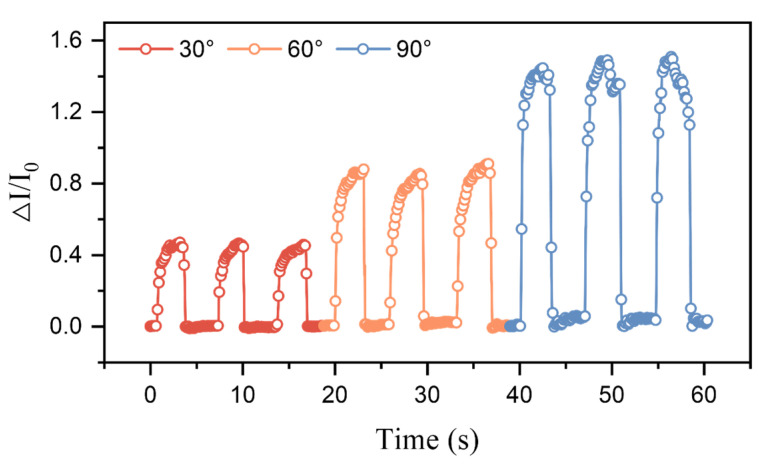
Testing of the ridge-mimicking spatially ordered flexible pressure sensor at different bending angles.

The ridge-mimicking spatially ordered microstructure sensor developed in this study exhibits high sensitivity and rapid response, enabling real-time monitoring of dynamic pressure changes. As shown in Figure 7, the sensor was attached to the index finger joint, where variations in the signal were used to detect the degree of finger bending by correlating pressure changes with the corresponding current signals. Significant changes in the current were observed when the index finger bent at angles of 15°, 30°, 60°, and 90°, allowing for accurate identification of the bending angle. Multiple measurements at the same angle demonstrated good consistency, as shown in Figure 8. The tested results indicate that this flexible pressure sensor based on ridge-mimicking spatially ordered microstructures holds great potential not only for detecting pressure magnitudes but also for applications such as monitoring knee joint flexion, tracking movement states, and human–machine interactions.

## 4. Conclusions

In this study, we proposed a novel design strategy for flexible pressure sensors, incorporating gradient-varying ridge-mimicking spatially ordered microstructures. By employing P-type doped graphene and multi-walled carbon nanotubes as the base materials, direct laser writing was used to fabricate a gradient-varying pressure-sensitive layer through layer-by-layer scanning. This approach enables the efficient fabrication of flexible pressure sensors with conductive microcones, exhibiting varying pressure sensitivities. Performance testing revealed that the sensor achieved a sensitivity of 1.65 kPa^−1^ across a pressure range of 0 to 1100 kPa, with a linearity (R^2^) of 0.996, a response time of 62 ms, and a recovery time of 83 ms. Moreover, the sensor maintained high sensitivity and fast response times across a broad pressure range. In finger bending experiments, the flexible pressure sensor effectively detected pressure magnitudes and bending angles with high repeatability. Compared to the other sensors, this sensor offers superior sensitivity, faster response times, and lower fabrication costs, making it highly promising for applications in smart robotics, wearable health devices, and human–machine interaction systems.

## Figures and Tables

**Table 1:** Sources of various materials and reagents.

Name	Purity/Specification	Manufacturer
MWCNTs	XFM13	XFNANO Materials, Nanjing, China
Graphene powder	G299147-100g	Aladdin Biochemical Technology, Shanghai, China
PDMS	DowCorning184	Dow Corning, Dayton, OH, USA
Anhydrous ethanol	Analytical Pure	Dayang Fine Chemicals, Shantou, China

**Table 2:** List of instruments and equipment.

Name	Model	Supplier
Electronic balance	BSA124S-CW	Sartorius, Göttingen, Germany
High-precision stepper motor	FSK40	FuRong, Shenzhen, China
Pressure testing machine	HYBH-101	Hengyuan, Yangzhou, China
Ultrasonic cleaner	SK5210LHC	Kudos Ultrasonic Instrument, Shanghai, China
Magnetic stirrer	90-2	Yuexin Instrument Manufacturing, Changzhou, China
Desktop spin coater	KW-4A	Institute of Microelectronics of the Chinese Academy of Sciences, Beijing, China
Magnetron sputtering coater	Explorer 14	Denton Vacuum, Moorestown, NJ, USA
Vacuum drying oven	DZF-6020	Yashilin Testing Equipment, Beijing, China
UV laser	SEAL-335-10S	Waner, Xiamen, China
Optical microscope	AO-HK830-5870T	Aosvi, Shenzhen, China
Data acquisition system	DAQ970A	Keysight Technologies, Santa Rosa, CA, USA
Digital source meter	Keithley2400	Keithley Instruments, Cleveland, OH, USA

**Table 3:** Comparison of piezoresistive sensors reported in the literature.

Electrodes and Dielectric Interface	Fabrication Method	Sensitivity (kPa^−1^)	Working Range (kPa)	Response Time (ms)	Recovery Time (ms)	References
Permeable conductive rubber/PDMS	Mold method	0.020~0.202	0.05~850	60	80	[12]
Silver nanoparticles/PDMS	Inkjet printing technique	0.48	0~15	250	250	[18]
Carbon composite conductors/PDMS	T-CVD method	0.3	0~5	162	108	[19]
MWCNTs/TPU	Injection compression molding	0.143	3~200	150	110	[20]
Conductive silver/composite ink	3D printing	1.22	2.8~8.1	/	/	[21]
ITO/PET thin film/PDMS	Mold method	0.094	10~150	105	194	[22]
Carbon/Graphene Fibers/Polymer	Hydrothermal method	0.45	0~500	92	26	[23]
Interdigitated electrodes	Direct laser writing	1.65	0~1100	62	83	This work

## Data Availability

Data will be made available on request.

## Supplementary Materials

The following supporting information can be downloaded at: https://www.mdpi.com/article/10.3390/mi15121441/s1, Figure S1. (a) Microstructure image of the conductive microstructure array (3 × 3 array). Height distribution of the microstructures at laser scanning speed of (b) 1000 mm/s, (c) 800 mm/s, (d) 1200 mm/s, (e) 1400 mm/s, (f) 1600 mm/s. Figure S2. Microstructure image of the conductive microstructure arrays (a) 2 × 2 array, (b) 4 × 4 array. Figure S3. (a) Sensitivity testing of the ridge-mimicking flexible pressure sensor at different scanning rate. (b) Sensitivity testing of the ridge-mimicking flexible pressure sensor with different microstructure arrangements.
