# Supplementary material for: Laser Fabrication and Performance of Flexible Pressure Sensors with Ridge-Mimicking Spatially Ordered Microstructures
Source: Micromachines (Basel). 2024 Nov 28;15(12):1441. doi: 10.3390/mi15121441 (PMC11677804; doi:10.3390/mi15121441)
Supplement: Supplementary file 1 [file micromachines-15-01441-s001.zip › micromachines-3286571-supplementary.pdf]

Supporting information

Laser Fabrication and Performance of Flexible Pressure Sensor with Ridge-Mimicking Spatially Ordered Microstructures

To investigate ridge-mimicking spatially ordered microstructures with different structural characteristics, we systematically investigated the effect of structure size on sensor sensitivity. Firstly, the height distribution of the ridge-mimicking microstructures is evaluated at different laser scanning speed, as shown in Figure S1. The results show that the faster the scanning speed, the lower the height of the microstructure (Figure S1(b)-(f)). The increase of the height of the single micro-cone provides more deformation space for the microstructures, which is beneficial to improve the sensitivity of the sensor, as shown in Figure S3(a). As the scanning speed increases, the height of the micro-cone decreases, and the sensitivity of the sensor decreases. However, as the height increases, the conductive micro-cone array has problems such as sudden response, and the conical microstructure is prone to bending deformation. Specifically, when the scanning speed is 800 mm/s, although the height of the microstructure is the highest, it has a sudden change in the response during the stress stage, and its sensitivity and linearity are lower than the sensitivity when the scanning speed is 1000 mm/s. Therefore, the subsequent sensor uses a scanning speed of 1000 mm/s.

Additionally, the ridge-mimicking microstructures with different arrangement are investigated, as show in Figure S2. To compare morphology of the original microstructures (3×3 array), the different microstructures of 2×2 array and 4×4 array are further prepared (Figure S2(a) and (b)). From the Fig. S2(a) and Fig. S3(b), it can be seen that when the microstructure arrangement is 2×2 array, the microstructure has little effect on the sensitivity of the sensor due to the less microstructure effect. When the microstructure arrangement is 4×4 array, because the micro-cone structure is too dense, when the sensor is stressed, multiple micro-cones are stressed together, which reduces the deformation of the micro-cone and reduces the sensitivity of the sensor. When the microstructure arrangement is 3×3 array, the microstructure density is more reasonable and the sensitivity is the highest, thus the subsequent sensor adopts the microstructure of 3×3 array.

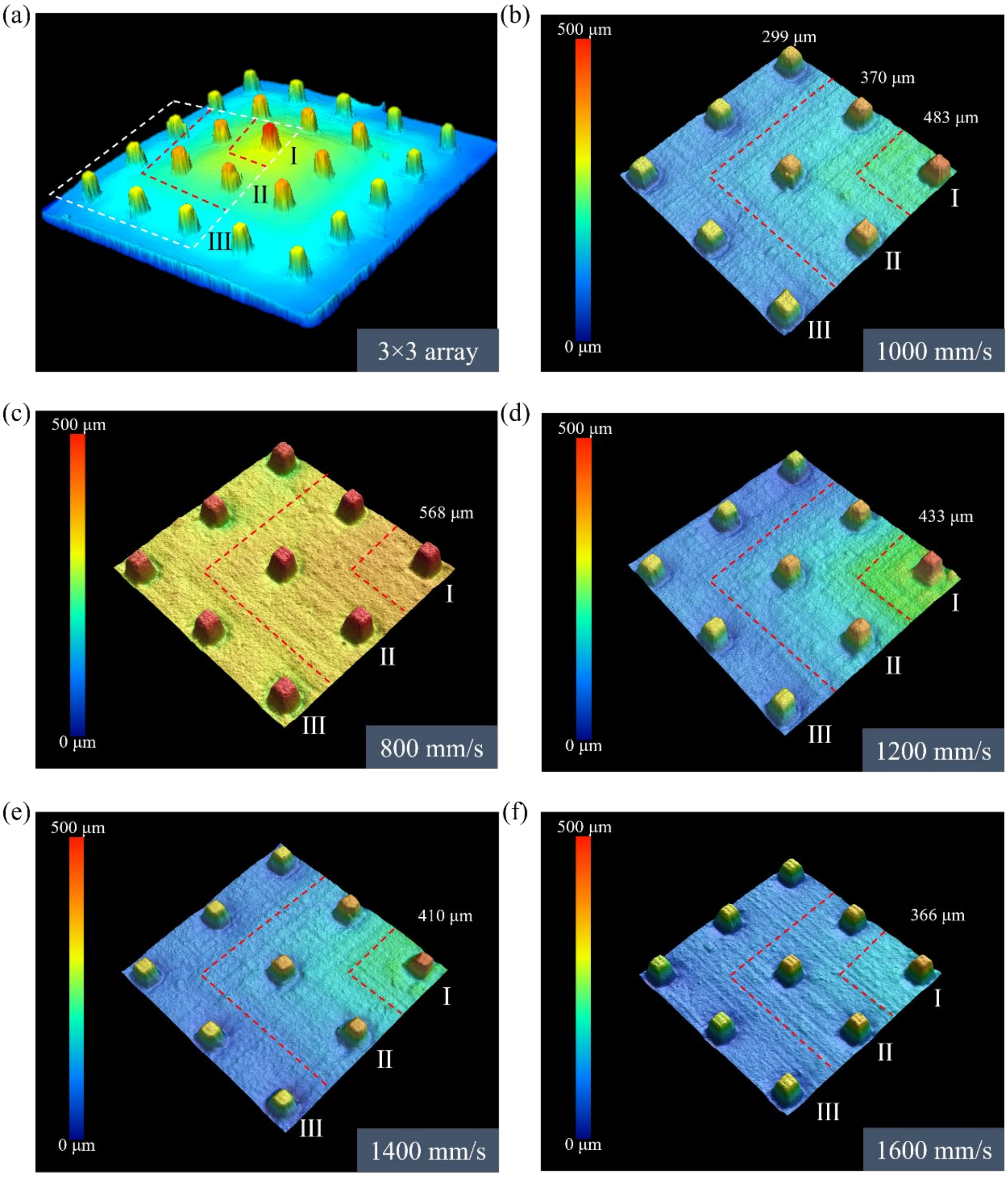

Figure S1 (a) Microstructure image of the conductive microstructure array (3×3 array). Height distribution of the microstructures at laser scanning speed of (b) 1000 mm/s, (c) 800 mm/s, (d) 1200 mm/s, (e) 1400 mm/s, (f) 1600 mm/s.

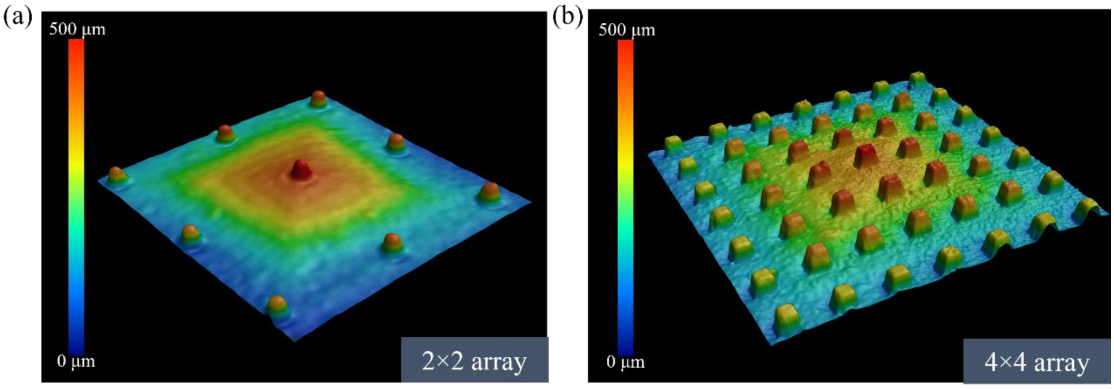

Figure S2 Microstructure image of the conductive microstructure arrays (a) 2×2 array, (b) 4×4 array.

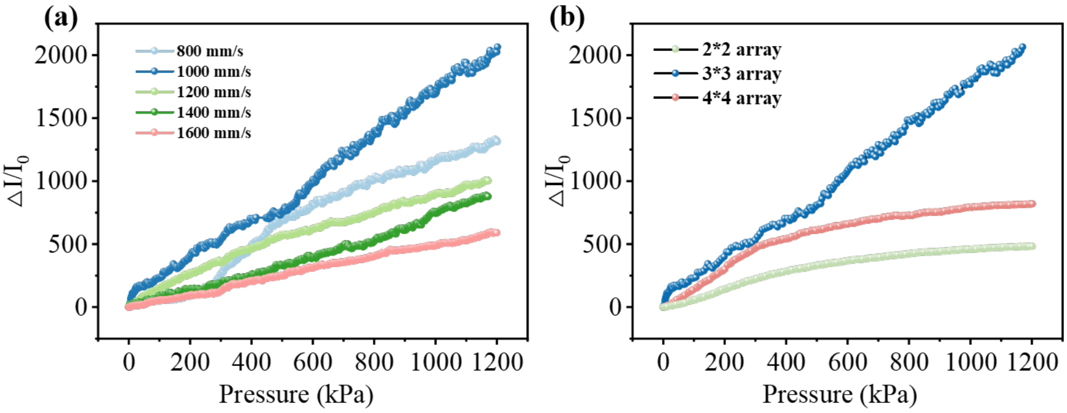

Figure S3 (a) Sensitivity testing of the ridge-mimicking flexible pressure sensor at different scanning rate. (b) Sensitivity testing of the ridge-mimicking flexible pressure sensor with different microstructure arrangements.
